# Leveraging risk maps of malaria vector abundance to guide control efforts reduces malaria incidence in Eastern Province, Zambia

**DOI:** 10.1038/s41598-020-66968-w

**Published:** 2020-06-25

**Authors:** David A. Larsen, Anne Martin, Derek Pollard, Carrie F. Nielsen, Busiku Hamainza, Matthew Burns, Jennifer Stevenson, Anna Winters

**Affiliations:** 1 Syracuse University, Syracuse, NY USA; 2 Akros Research, Lusaka, Zambia; 3 US President’s Malaria Initiative, US Centers for Disease Control and Prevention, Atlanta, GA USA; 4 Zambia National Malaria Elimination Program, Lusaka, Zambia; 5 Macha Research Trust, Choma, Zambia; 6 Johns Hopkins Malaria Research Institute, Baltimore, MD USA; 7 University of Montana, Missoula, MT USA

**Keywords:** Ecological epidemiology, Malaria

## Abstract

Although transmission of malaria and other mosquito-borne diseases is geographically heterogeneous, in sub-Saharan Africa risk maps are rarely used to determine which communities receive vector control interventions. We compared outcomes in areas receiving different indoor residual spray (IRS) strategies in Eastern Province, Zambia: (1) concentrating IRS interventions within a geographical area, (2) prioritizing communities to receive IRS based on predicted probabilities of *Anopheles funestus*, and (3) prioritizing communities to receive IRS based on observed malaria incidence at nearby health centers. Here we show that the use of predicted probabilities of *An. funestus* to guide IRS implementation saw the largest decrease in malaria incidence at health centers, a 13% reduction (95% confidence interval = 5–21%) compared to concentrating IRS geographically and a 37% reduction (95% confidence interval = 30–44%) compared to targeting IRS based on health facility incidence. These results suggest that vector control programs could produce better outcomes by prioritizing IRS according to malaria-vector risk maps.

## Introduction

Infectious disease frequently clusters within populations. An estimated 20% of the population suffers from 80% of infectious diseases^1^. When this heterogeneity is due to behaviors or characteristics of populations, public health interventions can be targeted based on those behaviors or population characteristics^2^. For example, vaccinating communities near incident smallpox cases led to smallpox elimination in Western Africa despite smallpox region-wide vaccination coverage not exceeding 80% of the population^3,4^. For vector-borne illnesses such as malaria, the heterogeneity in risk can be explained at least in part by the spatial heterogeneity of vector habitat^5,6^, and that heterogeneity in risk can be visualized through risk mapping to aid public health initiatives

The earliest malaria risk maps date back to the 1960’s, when Soviet epidemiologists classified the globe into zones of malaria transmission intensity^7^. With advances in spatial methods and computing power, the 21^st^ century has seen a proliferation of malaria risk maps with at least one risk map for nearly every malaria-endemic country and a variety of different measures of risk^8^. Still, these maps are not typically used to determine intervention allocation below the district or county level^8^. For example in Tanzania, malaria vector control coverage was alarmingly lower in areas at higher risk of malaria transmission due to those areas’ tendency to be more remote^9^.

Although it is accepted that certain areas have higher rates and risk of malaria transmission, these facts are not operationalized into malaria control campaigns. The insecticide-treated mosquito net (ITN) is most often hailed as a universal vector control intervention, and widespread scale-up of ITN coverage across sub-Saharan Africa is credited with saving millions of lives in the early 21^st^ century^10–12^. Due to its much higher cost^13^, indoor residual spray (IRS) is not nearly as widespread as ITNs^14^. Limited and declining operational funding available for IRS^15^, allows for an examination of how malaria risk maps can be operationalized for precision public health delivery. Without the capacity to spray all the houses at risk of malaria, risk maps could potentially be leveraged to prioritize areas to receive IRS intervention.

The current paradigm of large IRS operations is to concentrate IRS within a geo-political area such as a district^15^, while not spraying any houses in neighboring districts. The district to receive IRS is typically selected because it reports a high burden of malaria, but this approach may run contrary to the spatial heterogeneity of malaria transmission within districts. Instead of targeting all houses within a selected district, should the resources be spread throughout districts based on a measure of malaria burden and spraying as many houses as funding allows? And, if programs do spread resources across geo-political units, how should programs do so?

To address these questions, we compared outcomes of different IRS implementation strategies in Zambia. Six districts in Eastern Province, Zambia, were divided into three IRS implementation strategies: two districts sprayed IRS in as many communities as possible without prioritizing communities based on risk; two districts prioritized IRS in communities according to predicted *Anopheles funestus* density^16^, and two districts prioritized IRS in communities according to the observed malaria incidence at health facilities^17^. While operational budgets allowed for spraying an estimated 50% of the structures across these districts, communities targeted for IRS operations sought to achieve >90% coverage in accordance with WHO IRS guidelines^18^.

## Methods

### Setting

**Figure 1:**
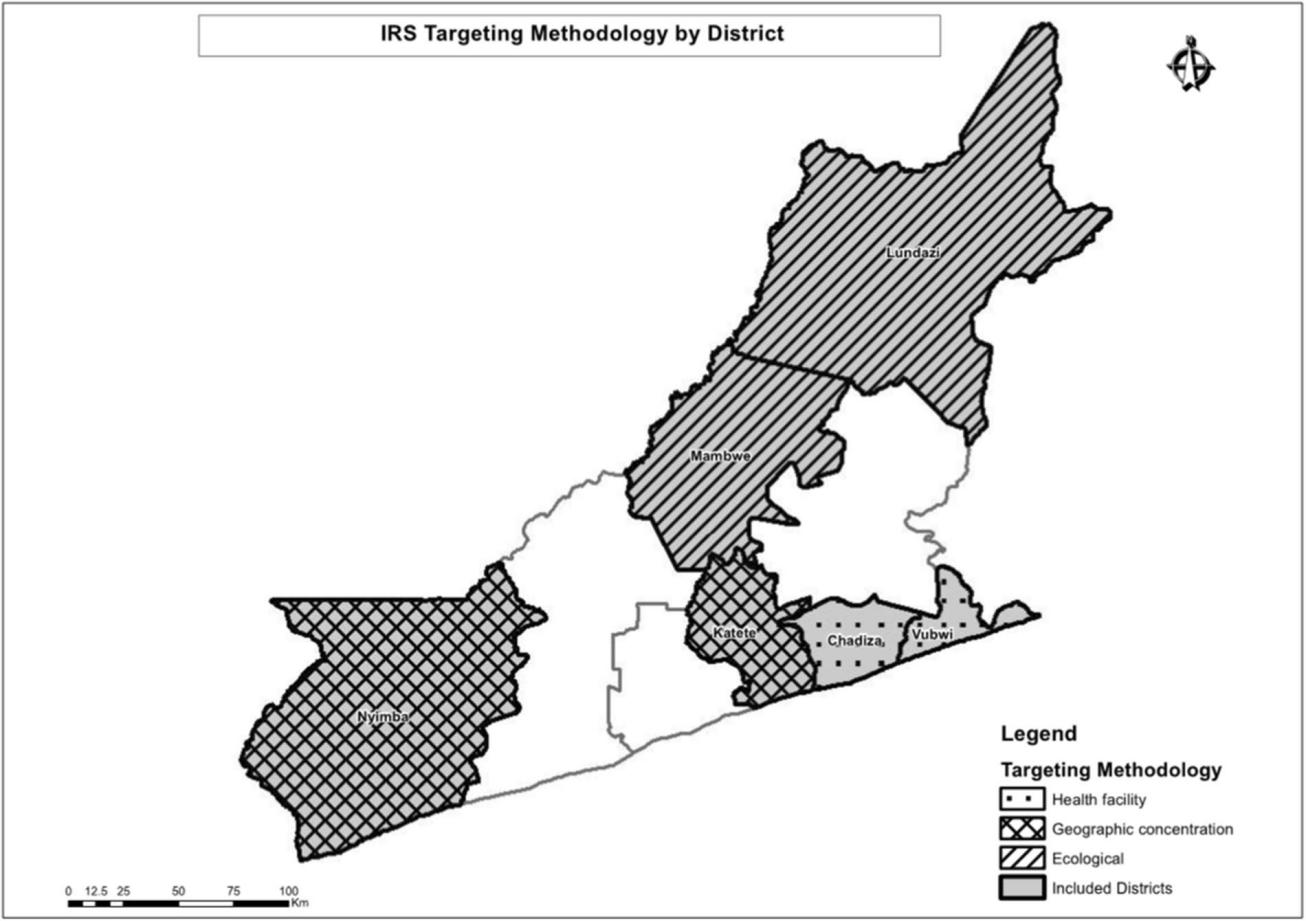
A map of the study area showing the allocation of different spray strategies.

The trial was conducted in Eastern Province, Zambia during the 2017 spray season (October to December 2017). Six largely rural districts in Eastern Province were included in the trial (Fig. 1). In these districts, *Anopheles funestus* and *An. gambiae* are the primary malaria vectors. Insecticide resistance to pyrethroids is widespread, and resistance to dicholorodiphenyltrichloroethane (DDT) and carbamates has been detected^19^. Malaria parasite prevalence has decreased in Eastern Province over the last 10 years, from 21% during the 2006 Malaria Indicator Survey (MIS)^20^ to 13% during the 2015 MIS^21^. ITNs are the primary vector control strategy and coverage of at least one ITN per household in Eastern Province was found to be less than 70% during MIS of 2008, 2010, 2012, and then at 93.8% in 2015^21–24^. IRS by government campaigns was first introduced in 2009, with 14% of houses in Eastern Province sprayed according to the 2010 MIS^23^ and 54.6% of houses sprayed according to the 2015 MIS^21^. In 2014 IRS operations began broadly using the organophosphate insecticide pirimiphos-methyl (Actellic®300CS, Syngenta). In Zambia, malaria testing and treatment at public health facilities is free for anyone attending. From surveys, others have estimated that nationwide approximately half of children with a fever will seek treatment from a health center where an estimated 70% of them receive a laboratory test for a malaria infection^25^.

### Study design

We compared outcomes of three different IRS prioritization strategies (described hereafter) using differences-in-differences comparison. Confirmed malaria incidence at the health center and reported to the health management information system (HMIS) served as the primary outcome and was available both before and after 2017 IRS operations. Entomological collections were conducted in the intervention arms after IRS implementation in 2017 and allowed for a post-only comparison.

### Indoor residual spraying

IRS was implemented in the study districts with funding from the President’s Malaria Initiative (PMI) and in accordance with the Zambian Ministry of Health/National Malaria Elimination Program and PMI Africa Indoor Residual Spraying (AIRS) program standards supplemented with mSpray, which is described in detail elsewhere^17,26–28^. IRS was conducted using pirimiphos-methyl (Actellic 300 CS). The funding for spray operations was independent of the trial; study investigators only served in an advisory capacity to actual implementation of the intervention. IRS in Eastern Province was implemented during November and December 2017, the months preceding heavy rains. According to MIS data previous campaigns have covered around 50% of total structures within Eastern Province (with higher coverage of structures targeted), and we expected a similar level of coverage within trial districts.

### IRS targeting

**Figure 2:**
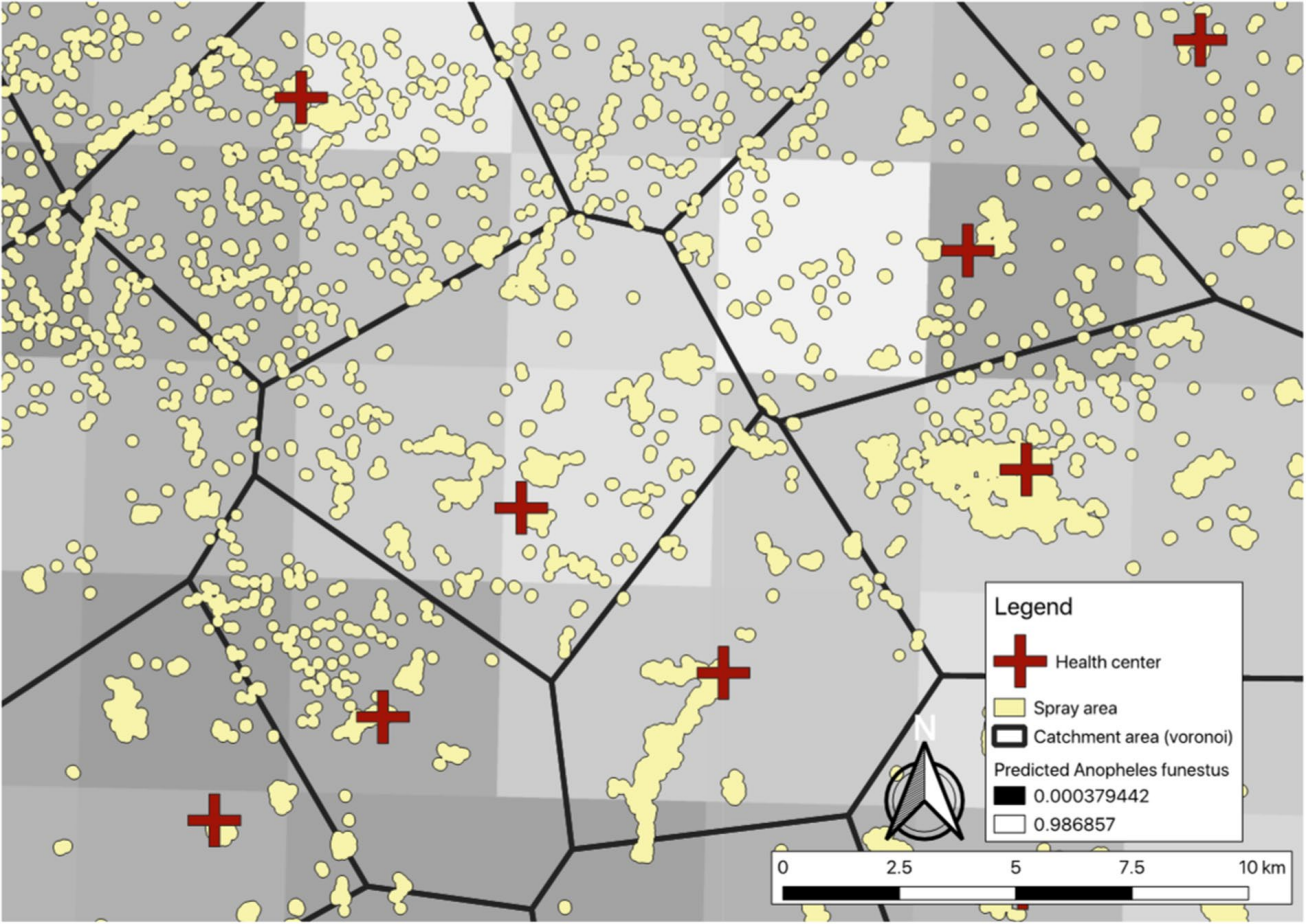
Zoom-level view of communities and different IRS allocation strategies. When allocating based on health facility-level incidence, communities within catchment areas would be sprayed or not, depending on the numbers of cases per the nearest health center. When allocating based on predicted *An. funestus* habitat, communities within lighter squares would be sprayed or not. Sub-geographic concentration would be to spray all communities within an arbitrary boundary.

Three different spray allocation strategies were deployed through the trial: sub-geographic concentration (comparison arm), health facility targeting, and ecological targeting (Fig. 2).

#### Sub-geographic concentration - comparison arm

In the comparison arm, IRS was implemented in a fashion similar to many implementations across Africa, where the goal is to achieve heavy saturation or “blanket spraying” of IRS within a defined geographic area, often an administrative unit. In Zambia, unlike the majority of PMI-supported countries, all districts receive some amount of resources for spraying and withdrawing spray operations from one district to concentrate resources in another district was not feasible. Instead, to simulate a blanketed geographic area, we utilized a circular window within each district, beginning at the geographic center of the district and sprayed expanding outward until exhausting available operational funds.

#### Health facility targeting

Within the health-facility targeting arm of the trial, the decision where to apply IRS within a district was determined based on reported health facility malaria incidence in the HMIS over the previous two years (2016 and 2017 malaria transmission seasons) as detailed elsewhere^17^. Communities within a district were assigned to the nearest health center using Euclidean or linear distance, and a measure of malaria incidence was estimate for each community. Those with the highest estimated malaria incidence were prioritized for IRS. We selected Chadiza and Vubwi Districts into this arm due to them having the greatest variance in our measure of incidence of the six districts included in the trial.

#### Ecological targeting

In the ecological-focused arm of the trial, IRS was allocated based on predicted probability of *An. funestus* as developed by the Malaria Atlas Project (MAP)^29^. The median predicted probability of *An. funestus* for each community was extracted from the MAP estimates and used to prioritize communities for IRS. We selected Lundazi and Mambwe Districts into this arm due to them having the greatest variance in the measure of predicted *An. funestus* populations of the six districts included in the trial.

### Data collection and procedures

#### HMIS data

Monthly HMIS data, January 2014-May 2018, and by health facility, were retrieved from the DHIS2 for all of Eastern Province in August of 2018. Specific indicators used included laboratory-confirmed malaria cases (the majority of which are by rapid diagnostic test [RDT]), number of malaria tests conducted, monthly outpatient attendance, and annual health facility catchment area population.

#### Entomological data

Five CDC light traps were deployed monthly from December 2017 to April 2018 in each of the 25 villages purposefully selected by size (small and large) and spray status (sprayed or unsprayed) for entomological surveillance (5 villages per intervention district, excluding Katete which has ongoing entomological surveillance as part of AIRS operations). Five houses within each selected village were selected purposefully to ensure broad geographic coverage throughout the selected village. These houses then hosted the CDC light traps once a month for the duration of the entomological surveillance. Characteristics of participating households were collected, such as structure type, vector control interventions available, and occupancy. Additionally, indoor resting mosquitoes were collected using the Prokopack handheld aspirator (Emory University, Atlanta, Georgia) in two houses per village^30^. Aspirator collections commenced at 05:00am. Indoor walls, ceilings, the undersides of furniture, and hanging clothes were systematically aspirated using progressive down- and upward movements along their entire surface with a speed approximating 1 meter per second.

Traps and aspirator collections were processed within survey areas. Mosquitoes from both collection types were geo-referenced to the household in which they were collected and morphologically assigned to genus level and separated by sex. Samples were stored dry on silica and taken to Macha Research Trust, Choma District where they were morphologically assigned to species or species complex level using standard keys^31,32^, and then confirmed to species using PCR when appropriate.

#### Environmental data

We obtained estimates of environmental factors included in the adjusted analyses from Google Earth Engine. We retrieved normalized digital vegetation index (NDVI) from the Landsat Tier 1 8-day NDVI collection and aggregated to month (median) within Google Earth Engine. We retrieved precipitation from the Climate Hazards Group InfraRed Precipitation with Station data collection^33^ and aggregated to month (sum) within Google Earth engine. We retrieved yearly nighttime lights from the Visible Infrared Imaging Radiometer Suite^34^. And we retrieved elevation from a digital elevation model (DEM) created with the ASTER projection. We linked environmental values to each health facility in the HMIS using the Raster package^35,36^ in R version 3.5.1^37^. Health facility locations were buffered by 5,000 m and mean values of each raster within the buffer were assigned to each health facility. In cases of missing NDVI data due to high cloud cover, we used linear interpolation between nearest time points to impute data. For health facilities without geocoordinates (15%) we assumed the median value within the district of each environmental measure (monthly for NDVI and precipitation and yearly for nighttime lights).

#### ITN coverage

Information about ITN ownership was extracted from the 2018 Zambia MIS, which took place April–May 2018. The MIS over-sampled in Eastern Province by approximately 350 additional houses from both sprayed and unsprayed locations; which enabled a detection of 10-20% difference in ITN coverage in sprayed versus non-sprayed areas and between study arms.

### Sample size and statistical power for HMIS analysis

Data from all health centers in the six trial districts were included in the analyses of HMIS data. The primary outcome of confirmed malaria incidence allows for the estimation of power using formula provided by Hayes and Bennett^38^. The sample size calculations are based on the following parameters: 1) probability of committing a type-1 error of 5% (two-sided); 2) 80% statistical power to detect a difference in confirmed malaria incidence between trial arms, 3) an estimated catchment area population per health center of 10,000, 4) an estimated 3 malaria cases per person per year, and 4) a coefficient of variation between health facilities of 0.5. With these parameters, a minimum of 55 health centers per trial arm needed to be included to observe a 25% decrease in confirmed malaria incidence. Following the trial we conducted a post-hoc analysis of the coefficient of variation between health facilities within each trial arm.

### Analysis

#### HMIS data

We used a generalized linear model with the health center as a random intercept and a negative binomial link due to overdispersion in a difference-in-differences approach to assess the effect of different IRS targeting schemes on confirmed monthly malaria incidence. In the analysis we included the trial arm by time (pre- and post-) interaction and the health center population as the offset. We tested how well continuous environmental measures fit the data compared to those same measures categorized into quintiles using Aikake’s Information Criteria. We then adjusted with the following covariates: type of health facility (categorized into health post, health center, or hospital), NDVI lagged one month (categorized into quintiles), precipitation lagged one month (categorized into quintiles), yearly nighttime lights (continuous), altitude (categorized into quintiles), confirmed monthly malaria cases lagged one month, the number of confirmation tests performed that month (either RDT or microscopy), a continuous variable for time, and a Fourier term of time to account for seasonality^39^.

#### Entomological data

We used a generalized linear model with the data collection village as a random intercept and a negative binomial link due to overdispersion to model the total number of female *Anopheles* mosquitoes found at a household for each night of surveillance. In the analysis we included whether the village was sprayed or unsprayed, and tested for an interaction between receiving IRS and trial arm. We then adjusted for the following covariates: type of eaves (open or closed), ITN in the household (at least one hanging or not), type of collection method (light trap or prokopack), NDVI lagged one month (continuous), precipitation lagged one month (continuous), night light in 2018 (continuous), altitude (continuous), and month of the year (categorical).

### Ethics approval and consent to participate

This trial was reviewed and approved by the National Health Research Science council of the Ministry of Health in Lusaka, Zambia; ERES Converge IRB in Lusaka, Zambia (protocol #2017-Apr-004); and Syracuse University IRB in New York, USA (protocol #17-074). The United States Centers for Disease Control and Prevention reviewed and approved the protocol under Cat IIIB (CGH tracking #2017-241). Study methods were performed in accordance with the guidelines and regulations stipulated by these institutions and informed consent was obtained for all human data collections.

## Results

### Spray coverage and targeting adherence

Spray operations targeted 2,083 communities across the six districts in 2017. Of these, 1,670 were primary targets based on the respective targeting methods in each district and 409 communities were secondary or “buffer” targets, to be sprayed if all primarily targeted areas were sprayed with additional time and resources remaining. The median spray area size across all districts was 21 structures. Spray area size was notably larger in Mambwe and Vubwi districts as compared to the other districts’ spray areas (Table 1).

**Table 1:** Number of spray areas targeted and average spray area size across trial arm districts.

District	Trial arm	Number of spray areas targeted (buffer)	Total number of spray areas in district	Median number of houses per spray area	Mean number of houses per spray area (standard deviation)
Chadiza	Health facility-targeting	232 (41)	347	17	57.37 (115.33)
Katete	Geographic concentration	148 (40)	474	19	103.53 (411.23)
Lundazi	Ecological-targeting	988 (208)	1,695	19	32.75 (133.67)
Mambwe	Ecological-targeting	85 (15)	157	26	108.82 (334.34)
Nyimba	Geographic concentration	112 (99)	242	16.5	63.52 (177.02)
Vubwi	Health facility-targeting	105 (1)	142	29	74.01 (119.86)
Total		1,670 (409)	3,057	10	54.78 (217.34)

According to 2018 MIS data, there was no difference in IRS coverage across trial arms, with an estimated 60% of structures across the trial area receiving IRS (95% confidence interval = 45–74%). Adherence to the targeting strategy was high across all districts. The majority of houses sprayed were in the targeted areas, some in the secondarily targeted or buffer areas sprayed, and few non-targeted houses sprayed (Table 2). Spray coverage within sprayed communities was also high, typically exceeding 75% of houses targeted (Table 3).

**Table 2:** Targeted spray area adherence by district and trial arm.

District	Trial arm	Percentage of structures sprayed that were targeted	Percentage of structures targeted (primarily) that were sprayed
Chadiza	Health facility-targeted	99.1%	90.0%
Katete	Geographic concentration	96.1%	66.7%
Lundazi	Ecological-targeted	95.5%	78.8%
Mambwe	Ecological-targeted	99.3%	86.5%
Nyimba	Geographic concentration	98.0%	94.2%
Vubwi	Health facility-targeted	92.1%	90.1%

**Table 3:** Spray coverage within sprayed communities by district and trial arm.

District	Group	Spray coverage < 25%	Spray coverage 25–50%	Spray coverage 50–75%	Spray coverage 75–100%	Total communities sprayed
Chadiza	Health Facility	0	2	9	261	272
Katete	Blanket	0	11	18	125	154
Lundazi	Ecological	7	39	71	871	988
Mambwe	Ecological	0	0	2	97	99
Nyimba	Blanket	0	2	1	180	183
Vubwi	Health Facility	1	0	4	87	92
Total		8	54	105	1,621	1,788

### Health management information systems data analysis

**Figure 3:**
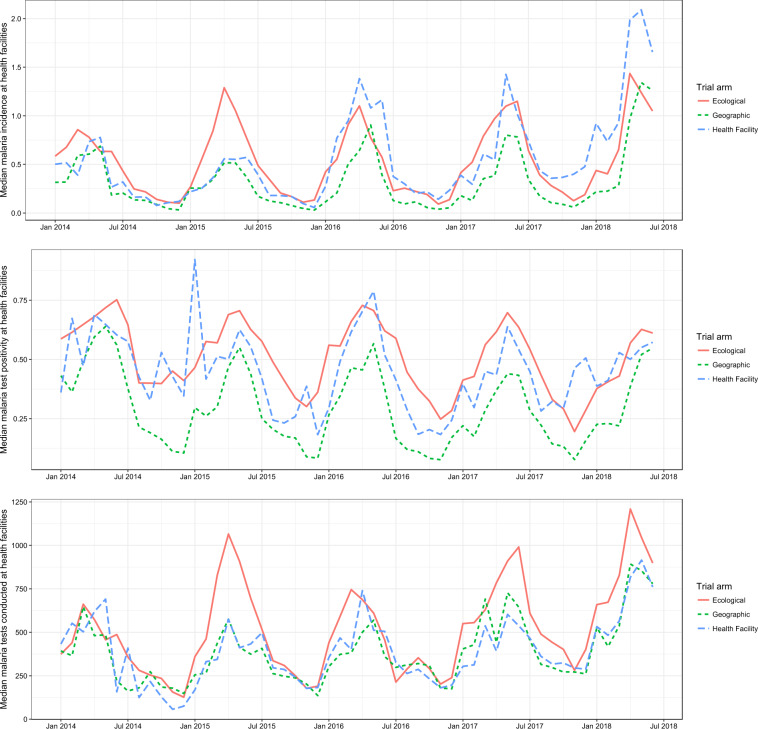
Unadjusted median trends of various malaria indicators at health facilities in the study area over time.

**Figure 4:**
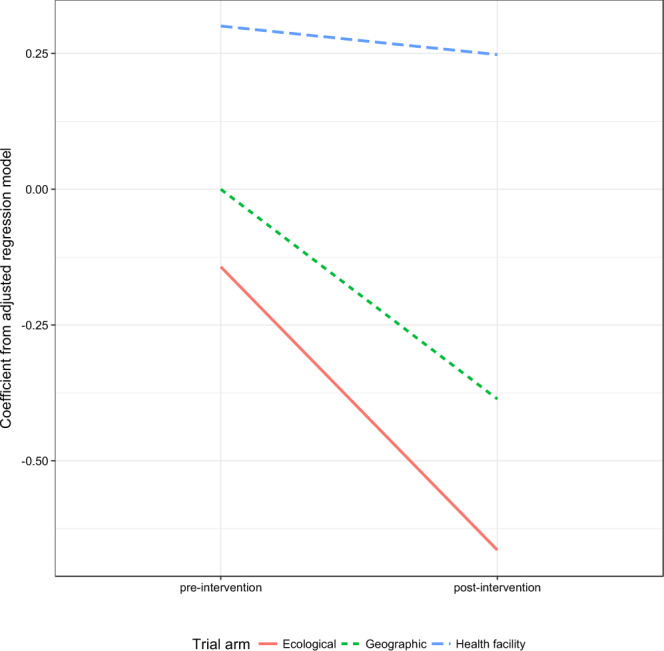
Adjusted coefficients of difference-in-differences analysis showing change in malaria incidence between pre- and post- periods and by trial arm.

A total of 55 facilities were included in the geographic-concentration arm, 34 facilities were included in the health facility targeting arm, and 69 facilities were included in the ecological targeting arm. Unadjusted confirmed malaria incidence appeared to increase throughout the study area 2014-2018, however the number of malaria confirmation tests performed (RDT or microscopy) also increased during this time period, and malaria test positivity was either stable or decreasing (Fig. 3). Before the 2017 IRS season, adjusted malaria incidence was highest in the health facility targeted arm, and lowest in the ecologically targeted arm (Fig. 4). Following IRS in 2017, adjusted malaria incidence decreased in all three arms, with the greatest decrease observed in the ecologically targeted arm (Fig. 4). The adjusted regression analysis showed that relative to the geographically-concentrated arm, confirmed malaria incidence decreased by 12.7% in the ecologically-concentrated arm following the 2017 IRS operations (incident-rate ratio [IRR] = 0.873, 95% confidence interval [CI] = 0.793–0.962) (Table 4). Also relative to the geographically-concentrated arm, confirmed malaria incidence increased by 39.6% in the health facility-targeted arm following the 2017 IRS operations (IRR = 1.396, 95% CI = 1.235–1.578) (Table 4). Environmental factors were associated with malaria incidence as expected (Table 4).

**Table 4:** Adjusted negative binomial regression predicting the outcome of confirmed malaria cases and using the estimated health facility catchment area population as the offset.

Factor	Categorization	IRR (95% CI)	p-value
Trial arm	Geographically-concentrated	Reference	Reference
	Health facility-targeted	1.350 (1.212–1.504)	<0.001
	Ecologically-targeted	0.867 (0.788–0.954)	0.004
Time period	Pre-2017 IRS operations	Reference	Reference
	Post-2017 IRS operations	0.680 (0.599–0.772)	<0.001
Trial arm by time period interaction	Geographically-concentrated by Post-2017 IRS operations	Reference	Reference
	Health facility-targeted by Post-2017 IRS operations	1.396 (1.235–1.578)	<0.001
	Ecologically-targeted by post-2017 IRS operations	0.873 (0.793–0.962)	0.006
Type of health facility	Health center	Reference	Reference
	Hospital	2.536 (1.836–3.503)	<0.001
	Health post	2.100 (1.956–2.255)	<0.001
NDVI lagged 1 month	Quintile 1 (0.148–0.205)	Reference	Reference
	Quintile 2 (0.205–0.275)	1.166 (1.089–1.248)	<0.001
	Quintile 3 (0.278–0.343)	2.055 (1.923–2.190)	<0.001
	Quintile 4 (0.362–0.430)	2.671 (2.515–2.837)	<0.001
	Quintile 5 (0.435–0.487)	2.759 (2.593–2.936)	<0.001
Precipitation lagged 1 month	Quintile 1 (0.0 mm–0.9 mm)	Reference	Reference
	Quintile 2 (0.9 mm–7.0 mm)	1.037 (0.978–1.098)	0.222
	Quintile 3 (7.0 mm–75.8 mm)	1.214 (1.147–1.284)	<0.001
	Quintile 4 (76.0 mm–183.2 mm)	1.262 (1.193–1.336)	<0.001
	Quintile 5 (183.2 mm–402.2 mm)	1.264 (1.189–1.344)	<0.001
Altitude	Quintile 1 (438 m–774 m)	Reference	Reference
	Quintile 2 (790 m–988 m)	0.706 (0.633–0.789)	<0.001
	Quintile 3 (988 m–1043 m)	0.498 (0.438–0.567)	<0.001
	Quintile 4 (1043 m–1125 m)	0.560 (0.505–0.621)	<0.001
	Quintile 5 (1128 m–1398 m)	0.543 (0.483–0.610)	<0.001
Yearly nighttime light	Continuous – increase of 1 on a scale from 0–63 (ranging from 0 to 2.0 in this dataset)	0.385 (0.330–0.448)	<0.001
Total confirmed malaria cases lagged 1 month	Continuous – increase of 100 cases	1.049 (1.045–1.053)	<0.001
Total malaria tests done (RDTs or microscopy)	Continuous – increase of 1000 tests	1.039 (1.034–1.045)	<0.001
Sine-function for time	Continuous	0.981 (0.919–1.047)	0.560
Cosine-function for time	Continuous	1.459 (1.319–1.615	<0.001
Time	Continuous – increase of one month	1.022 (1.016–1.029)	<0.001

N = 149 facilities, 5,587 months.

### Entomological collections analysis

A mean of 2.75 female *Anopheles* mosquitoes were collected per house per visit, although most collections did not report any *Anopheles* (56%). The majority of the *Anopheles* collected were not blood fed (90%), nor were they gravid (92%). Thirteen different *Anopheles* species were found; *An. funestus* was the most commonly collected *Anopheles* mosquito (47%), and *Anopheles arabiensis* was the second most commonly collected (18%). Female *Anopheles* of any species were less likely to be found in villages covered by IRS (IRR = 0.729, 95% CI = 0.581–0.913) (Table 5), and there was no evidence of interaction between IRS coverage and trial arm (likelihood ratio test = 3.07, p = 0.216).

**Table 5:** Adjusted negative binomial regression predicting the outcome of total *Anopheles* mosquitoes collected during entomological surveillance.

Factor	Categorization	IRR (95% CI)	p-value
Trial arm	Geographically-concentrated	Reference	Reference
	Health facility-targeted	1.020 (0.519–2.006)	0.954
	Ecological-targeted	1.915 (1.053–3.485)	0.033
IRS	No IRS in village	Reference	Reference
	IRS in village	0.729 (0.581–0.913)	0.006
Type of eaves	Closed	Reference	Reference
	Open	1.700 (1.375–2.103)	<0.001
ITN	None	Reference	Reference
	At least 1 hanging	0.923 (0.638–1.334)	0.669
Type of collection	Light trap	Reference	Reference
	Prokopack	0.197 (0.146–0.267)	<0.001
NDVI lagged 1 month	Continuous	0.355 (0.171–0.735)	0.005
Precipitation lagged 1 month	Continuous	1.001 (0.997–1.005)	0.764
Nighttime light	Continuous	0.655 (0.155–2.774)	0.566
Altitude	Continuous	1.000 (0.999–1.001)	0.521
Month	December	Reference	Reference
	January	1.825 (1.105–3.014)	<0.001
	February	2.544 (1.719–3.765)	<0.001
	March	4.409 (2.640–7.362)	<0.001
	April	7.373 (4.905–11.083)	<0.001

N = 25 sites, 863 houses.

Female *Anopheles* were twice as likely to be found in houses in the ecologically-targeted arm compared to the other two arms (Table 5). As expected, female *Anopheles* were more likely to be collected in houses with open eaves (IRR = 1.700, 95% CI = 1.375–2.103), and less likely to be collected with prokopacks than light traps (IRR = 0.197, 95% CI = 0.146–0.267) (Table 5). After accounting for the month that the collections took place, precipitation, nighttime light, and altitude were not associated with *Anopheles* counts, and increasing NDVI was associated with increasing numbers of mosquitoes collected.

### ITN coverage

An estimated 72% of individuals in the trial area slept under an ITN the previous night (95% CI = 67–78%). There was no evidence of difference between trial arms in ITN use.

### Post-hoc analysis of the coefficient of variation

The coefficient of variation of confirmed malaria incidence over the entire time period in the analysis, or *k* from Hayes and Bennett^38^, was estimated to be 0.70 for the geographic-concentration arm, 0.70 for the health facility targeted arm and 0.57 for the ecological targeting arm. These estimates did not change after the implementation of the targeting strategies.

## Discussion

These results indicate that the ecologically targeted arm of the trial saw the largest decrease in confirmed malaria incidence, 13% lower (95% confidence interval = 5–21%) than concentrating IRS geographically and 63% lower (95% confidence interval = 30–44%) than targeting IRS based on health facility incidence. These results are in agreement with some of our previous research in Zambia that found ecological measures more important for assessing risk than infected person measures^40^, as well as recent research showing that although hotspots may be dynamic they are still associated with proximity to vector habitat^41^.

Previous to this study, all health facilities across study districts saw a median 135 confirmed malaria cases each month (interquartile range 49–320) from 2014–2017. A thirteen percent reduction suggests that if employing an ecological targeting strategy rather than the more traditional geographic approach, health facilities would see 18 fewer cases per month or 216 fewer cases per year. Given that there were 158 health facilities in these districts, the 13% reduction in malaria incidence would be an estimated 34,128 fewer malaria cases each year seen at health centers. As perhaps only 50% of malaria cases are treated at health centers in Zambia^25^, the 34,000 fewer malaria cases are likely to underestimate the full impact of improved IRS delivery by using ecological targeting.

Numerous researchers have suggested that targeting malaria control to most affected areas is possible^5,6^. This is the first study of which we are aware that showed using ecological risk maps of vector abundance in the distribution of vector control interventions may be beneficial. A previous trial in Kenya found that targeting malaria control reduced malaria in serologically defined hotspots but not in the immediate vicinity of the area targeted^42^. Our entomological results suggest a similar mechanism occurring in this study, wherein malaria transmission was primarily affected in areas targeted for IRS. In this case, however we constructed the targeting strategy based upon availability of resources rather than with the hypothesis that we could impact transmission outside of targeting areas. Still, prioritizing areas with the highest number of vectors had the greatest impact.

Given that IRS is delivered to household locations, the logistical feasibility of employing an approach outlined herein should be considered. Two needs must play out for this to be feasible. First, significant political will must exist to shift what may be long standing practices in IRS implementation and distribution; some areas visited under the ecological model had not received spray in recent years, often for strategic reasons such as distance and being smaller in size than some easier to reach areas. Bringing IRS to new areas and removing IRS from areas that usually receive it, will require additional logistics and community mobilization. Community acceptance of IRS is a huge hurdle that programs face, and they may not be willing to move IRS from an area that is known to be receptive, to an area that has never received it and could potentially refuse. Second, identifying areas with this targeting method and then accurately delivering spray to the prioritized areas, requires detailed mapping and monitoring tools. The success of this trial was facilitated by mSpray, but not all IRS implementations invest in such spatial aids

**Figure 5:**
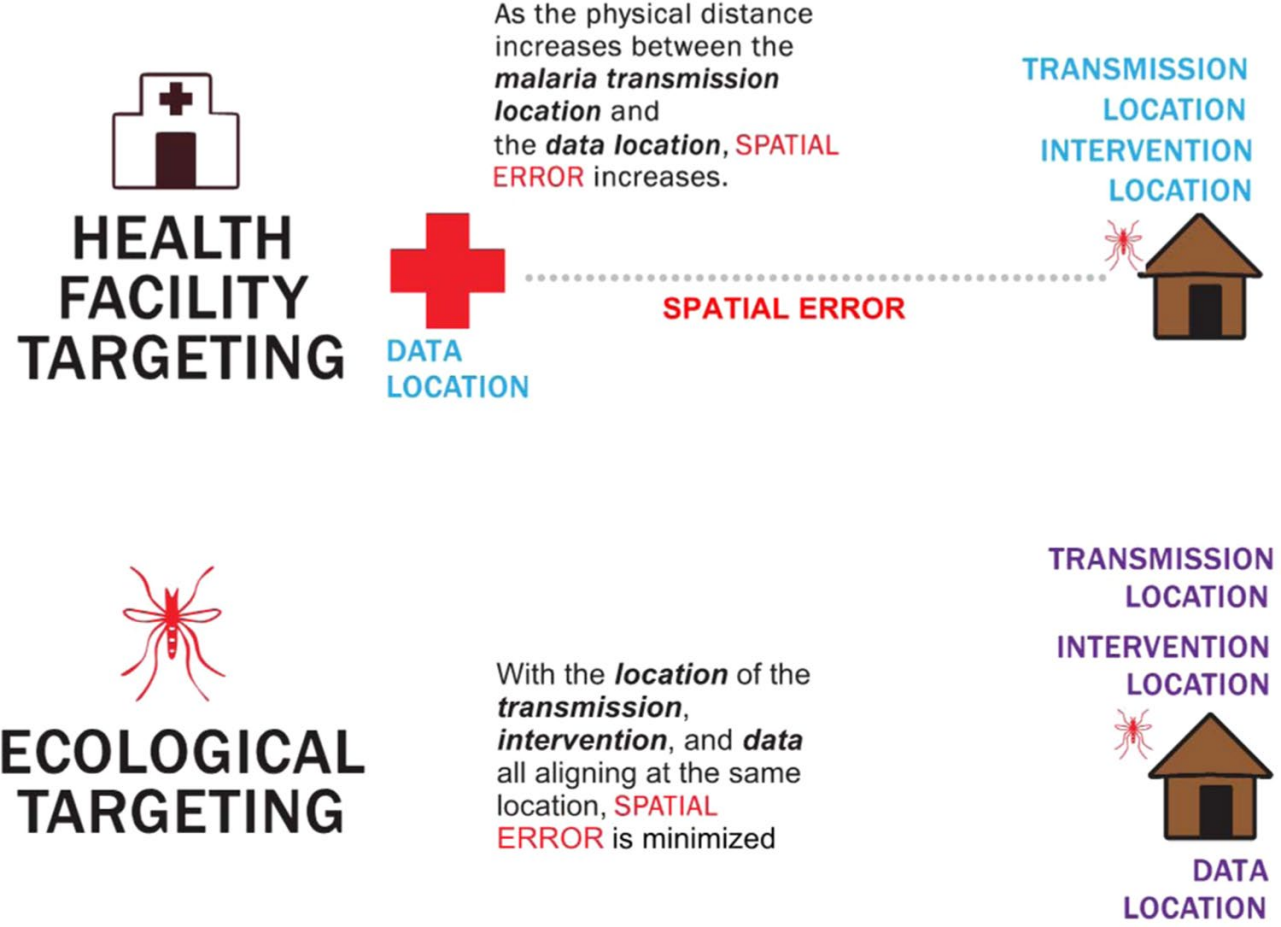
A diagram showing how spatial error differs when attributing risk to household locations.

Not all risk maps are equally valuable for targeting interventions and our results suggest that how targeting is conducted matters a great deal. In malaria transmission, the event of importance is the infective mosquito bite, which is most likely to occur where the vector is most abundant. In areas with high malaria incidence, ecological risk mapping of vector abundance best predicts the location of an infective bite. When coupled with satellite enumeration of housing, ecological risk maps aligns the probability of the infective bite with the location of the IRS implementation. On the other hand, using health facility incidence to determine which houses receive IRS implementation leverages the location of the treatment seeking rather than a probable location of an infective bite. Spatial error is greater when using incidence data aggregated to the location of the health facility than when using ecological risk maps (Fig. 5).

In the ecologically-targeted arm of this trial we used a global predicted habitat for the *Anopheles funestus* developed by the Malaria Atlas Project^29^. There were no locally-calibrated risk maps available for malaria vectors in Eastern Province, Zambia, which may have improved the results from ecological targeting in this case. These results should not be used to stifle the generation of locally-calibrated risk maps. Still, the use of a global map is beneficial because malaria control programs can adopt this type of IRS allocation even if they lack locally-specific risk maps or entomological data. Furthermore, these results validate the concept of using the Malaria Atlas Project vector probabilities map to guide vector control intervention prioritization to communities.

### Limitations

We acknowledge that the study design that we used is not ideal for assessing intervention delivery and therefore results should be interpreted with caution. Specifically, the different trial districts may differ in terms of malaria transmission intensity. The nature of the research question and available funding precluded a district-randomized trial, which if performed robustly would have encompassed half the country of Zambia. Theoretically the difference-in-differences design we utilized should account for these differences, and we included various environmental measures in the analysis to attempt to improve the comparison of the different districts. These environmental measures were highly associated with the outcome, and in the expected direction suggesting that our modeling of the health facility incidence improved the comparability of the districts. We also prescriptively allocated districts to different IRS prioritization strategies based on the variance of *An. funestus* and health facility malaria incidence. Prioritizing IRS delivery based on any parameter is not likely to be useful where those parameters do not vary geographically. Further, we used HMIS data to assess the difference between IRS strategies. These data are often incomplete and have numerous documented limitations^43^. We have attempted to account for numerous issues within the HMIS data by including measures in the models such as the number of confirmation tests done and the previous months’ malaria cases. The results we found were robust to these different measures, and so we are fairly confident in the modeling process. We do not expect the errors within the data to be associated with the spray strategy, therefore precluding bias in the analysis. Another limitation is that the ecologically-targeted arm was the most novel arm, being the most different from the way IRS was distributed in previous years. Perhaps the strategy may have been less important than the fact that teams sprayed houses that did not receive IRS in previous years. We consider this possibility unlikely, as the analysis incorporated both sprayed and unsprayed areas in each IRS prioritization strategy therefore including any malaria resurgence from changing spray operations that has been observed in other settings^44,45^.

## Conclusions

The use of ecologically-derived risk maps for malaria to guide allocation of IRS resources was associated with a 13% (95% confidence interval = 5–21%) decrease in malaria incidence compared to the traditional approach of geographically concentrating those resources. IRS programs may see increased impact if areas of greatest malaria vector abundance are targeted.

## Declarations

The findings and conclusions in this report are those of the authors and do not necessarily represent the official position of the United States Centers for Disease Control and Prevention.

### Consent for publication

Both the United States President’s Malaria Initiative and the Zambian National Health Research Authority reviewed the article before approving submission to a scientific journal. Due to Dr. Carrie Nielsen’s involvement, the manuscript also had to receive United States Centers for Disease Control and Prevention approval before being submitted for peer review.

## Data Availability

The HMIS and MIS data used in this analysis are proprietary data owned by the Zambian Ministry of Health, and request to access HMIS or MIS data used in this analysis can be made to the Zambian Ministry of Health. Entomological data generated from this trial are available from the corresponding author with reasonable request and required IRB approvals. All environmental data used in this study are freely available from Google Earth Engine.
